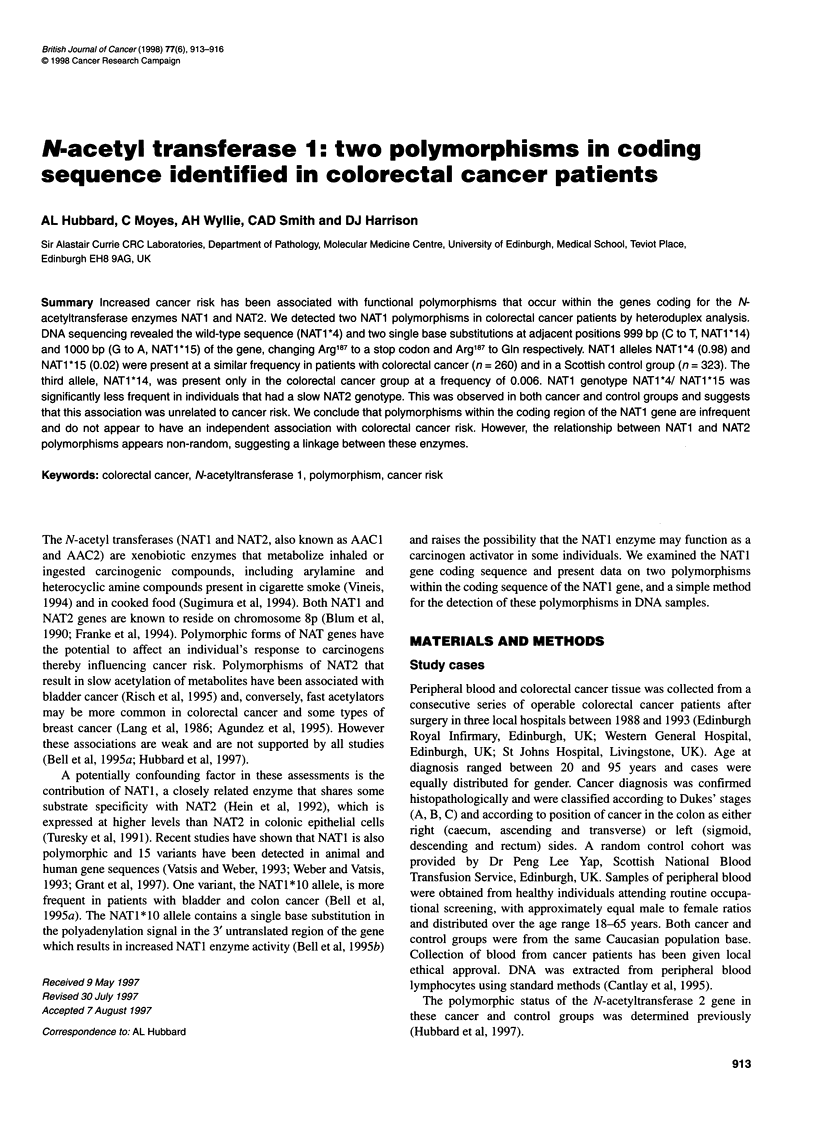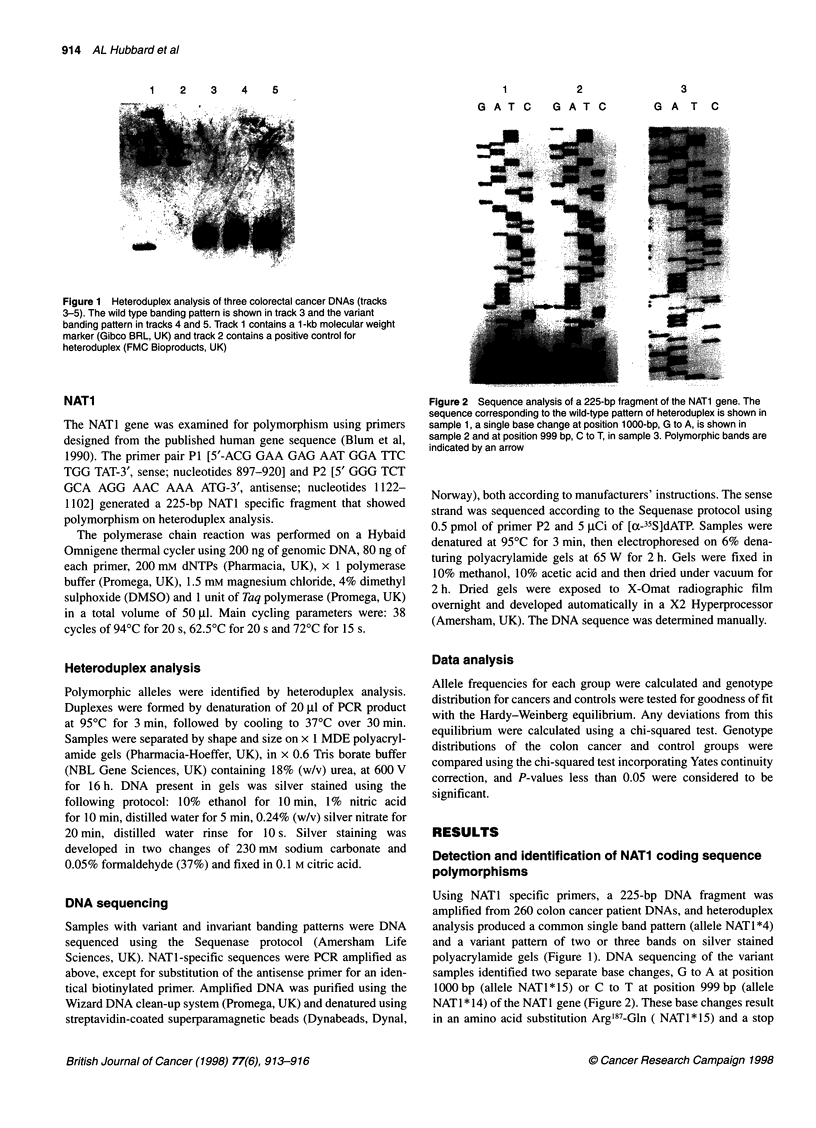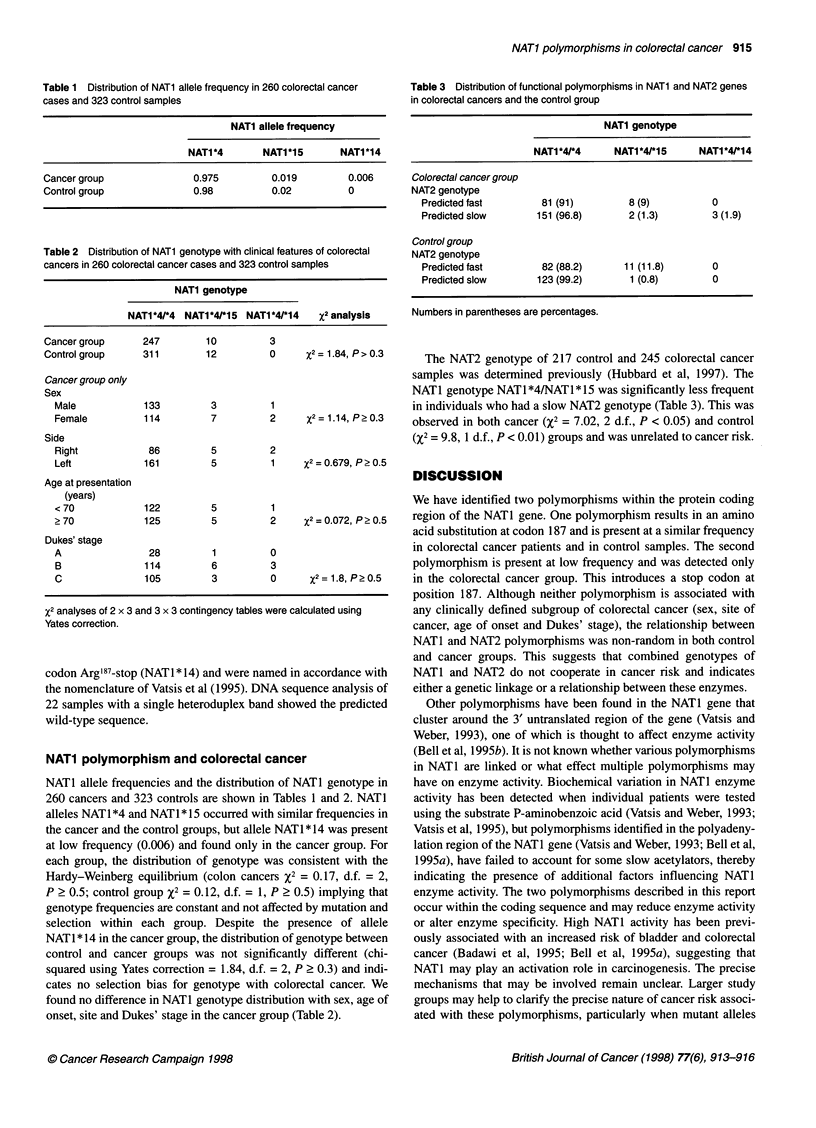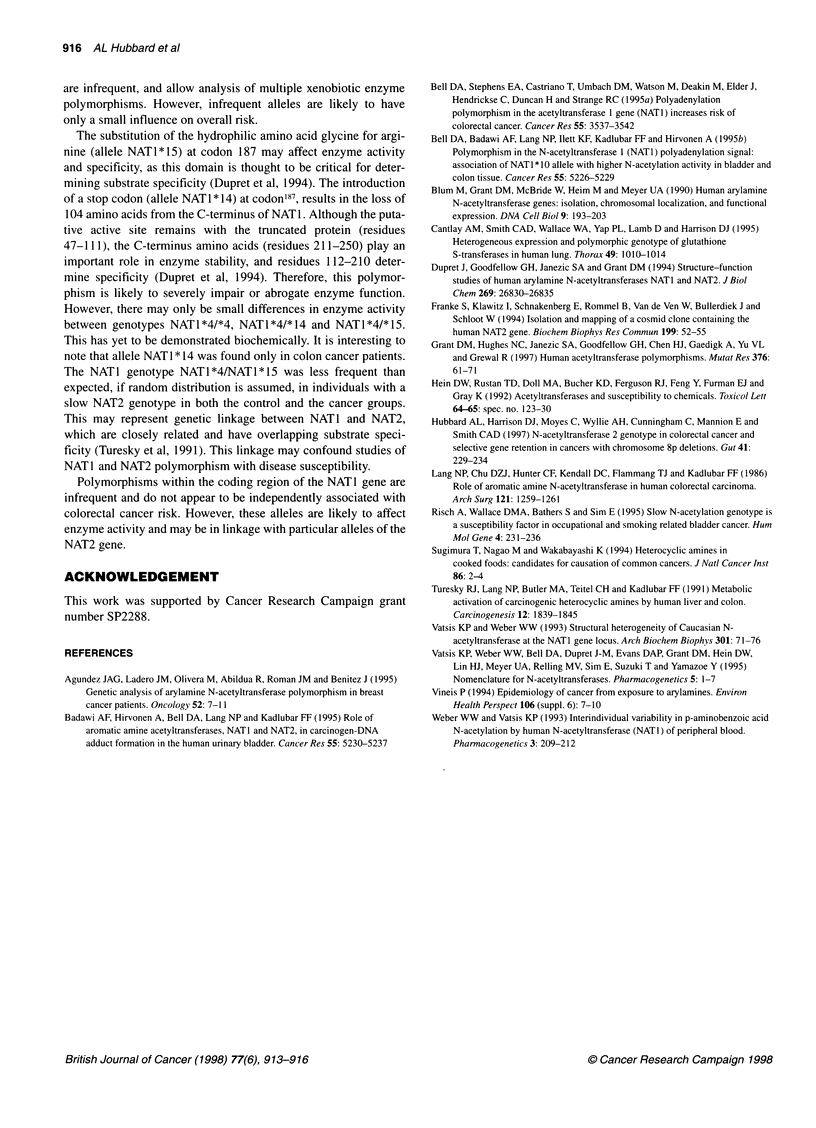# N-acetyl transferase 1: two polymorphisms in coding sequence identified in colorectal cancer patients.

**DOI:** 10.1038/bjc.1998.151

**Published:** 1998-03

**Authors:** A. L. Hubbard, C. Moyes, A. H. Wyllie, C. A. Smith, D. J. Harrison

**Affiliations:** Sir Alastair Currie CRC Laboratories, Department of Pathology, University of Edinburgh, Medical School, UK.

## Abstract

**Images:**


British Journal of Cancer (1998) 77(6), 913–916
© 1998 Cancer Research Campaign

### *N*-acetyl transferase 1: two polymorphisms in coding sequence identified in colorectal cancer patients

**AL Hubbard, C Moyes, AH Wyllie, CAD Smith and DJ Harrison**

Sir Alastair Currie CRC Laboratories, Department of Pathology, Molecular Medicine Centre, University of Edinburgh, Medical School, Teviot Place, Edinburgh EH8 9AG, UK

**Summary** Increased cancer risk has been associated with functional polymorphisms that occur within the genes coding for the *N*-acetyltransferase enzymes NAT1 and NAT2. We detected two NAT1 polymorphisms in colorectal cancer patients by heteroduplex analysis. DNA sequencing revealed the wild-type sequence (NAT1*4) and two single base substitutions at adjacent positions 999 bp (C to T, NAT1*14) and 1000 bp (G to A, NAT1*15) of the gene, changing $Arg^{187}$ to a stop codon and $Arg^{187}$ to Gln respectively. NAT1 alleles NAT1*4 (0.98) and NAT1*15 (0.02) were present at a similar frequency in patients with colorectal cancer ($n = 260$) and in a Scottish control group ($n = 323$). The third allele, NAT1*14, was present only in the colorectal cancer group at a frequency of 0.006. NAT1 genotype NAT1*4/ NAT1*15 was significantly less frequent in individuals that had a slow NAT2 genotype. This was observed in both cancer and control groups and suggests that this association was unrelated to cancer risk. We conclude that polymorphisms within the coding region of the NAT1 gene are infrequent and do not appear to have an independent association with colorectal cancer risk. However, the relationship between NAT1 and NAT2 polymorphisms appears non-random, suggesting a linkage between these enzymes.

**Keywords:** colorectal cancer, *N*-acetyltransferase 1, polymorphism, cancer risk

The *N*-acetyl transferases (NAT1 and NAT2, also known as AAC1 and AAC2) are xenobiotic enzymes that metabolize inhaled or ingested carcinogenic compounds, including arylamine and heterocyclic amine compounds present in cigarette smoke (Vineis, 1994) and in cooked food (Sugimura et al, 1994). Both NAT1 and NAT2 genes are known to reside on chromosome 8p (Blum et al, 1990; Franke et al, 1994). Polymorphic forms of NAT genes have the potential to affect an individual's response to carcinogens thereby influencing cancer risk. Polymorphisms of NAT2 that result in slow acetylation of metabolites have been associated with bladder cancer (Risch et al, 1995) and, conversely, fast acetylators may be more common in colorectal cancer and some types of breast cancer (Lang et al, 1986; Agundez et al, 1995). However these associations are weak and are not supported by all studies (Bell et al, 1995*a*; Hubbard et al, 1997).

A potentially confounding factor in these assessments is the contribution of NAT1, a closely related enzyme that shares some substrate specificity with NAT2 (Hein et al, 1992), which is expressed at higher levels than NAT2 in colonic epithelial cells (Turesky et al, 1991). Recent studies have shown that NAT1 is also polymorphic and 15 variants have been detected in animal and human gene sequences (Vatsis and Weber, 1993; Weber and Vatsis, 1993; Grant et al, 1997). One variant, the NAT1*10 allele, is more frequent in patients with bladder and colon cancer (Bell et al, 1995*a*). The NAT1*10 allele contains a single base substitution in the polyadenylation signal in the 3′ untranslated region of the gene which results in increased NAT1 enzyme activity (Bell et al, 1995*b*) and raises the possibility that the NAT1 enzyme may function as a carcinogen activator in some individuals. We examined the NAT1 gene coding sequence and present data on two polymorphisms within the coding sequence of the NAT1 gene, and a simple method for the detection of these polymorphisms in DNA samples.

*Received 9 May 1997*
*Revised 30 July 1997*
*Accepted 7 August 1997*

*Correspondence to:* AL Hubbard

### MATERIALS AND METHODS

### Study cases

Peripheral blood and colorectal cancer tissue was collected from a consecutive series of operable colorectal cancer patients after surgery in three local hospitals between 1988 and 1993 (Edinburgh Royal Infirmary, Edinburgh, UK; Western General Hospital, Edinburgh, UK; St Johns Hospital, Livingstone, UK). Age at diagnosis ranged between 20 and 95 years and cases were equally distributed for gender. Cancer diagnosis was confirmed histopathologically and were classified according to Dukes' stages (A, B, C) and according to position of cancer in the colon as either right (caecum, ascending and transverse) or left (sigmoid, descending and rectum) sides. A random control cohort was provided by Dr Peng Lee Yap, Scottish National Blood Transfusion Service, Edinburgh, UK. Samples of peripheral blood were obtained from healthy individuals attending routine occupational screening, with approximately equal male to female ratios and distributed over the age range 18–65 years. Both cancer and control groups were from the same Caucasian population base. Collection of blood from cancer patients has been given local ethical approval. DNA was extracted from peripheral blood lymphocytes using standard methods (Cantlay et al, 1995).

The polymorphic status of the *N*-acetyltransferase 2 gene in these cancer and control groups was determined previously (Hubbard et al, 1997).

**Figure 1** Heteroduplex analysis of three colorectal cancer DNAs (tracks 3–5). The wild type banding pattern is shown in track 3 and the variant banding pattern in tracks 4 and 5. Track 1 contains a 1-kb molecular weight marker (Gibco BRL, UK) and track 2 contains a positive control for heteroduplex (FMC Bioproducts, UK)

**Figure 2** Sequence analysis of a 225-bp fragment of the NAT1 gene. The sequence corresponding to the wild-type pattern of heteroduplex is shown in sample 1, a single base change at position 1000-bp, G to A, is shown in sample 2 and at position 999 bp, C to T, in sample 3. Polymorphic bands are indicated by an arrow

### NAT1

The NAT1 gene was examined for polymorphism using primers designed from the published human gene sequence (Blum et al, 1990). The primer pair P1 [5′-ACG GAA GAG AAT GGA TTC TGG TAT-3′, sense; nucleotides 897–920] and P2 [5′ GGG TCT GCA AGG AAC AAA ATG-3′, antisense; nucleotides 1122–1102] generated a 225-bp NAT1 specific fragment that showed polymorphism on heteroduplex analysis.

The polymerase chain reaction was performed on a Hybaid Omnigene thermal cycler using 200 ng of genomic DNA, 80 ng of each primer, 200 mM dNTPs (Pharmacia, UK), × 1 polymerase buffer (Promega, UK), 1.5 mM magnesium chloride, 4% dimethyl sulphoxide (DMSO) and 1 unit of *Taq* polymerase (Promega, UK) in a total volume of 50 µl. Main cycling parameters were: 38 cycles of 94°C for 20 s, 62.5°C for 20 s and 72°C for 15 s.

### Heteroduplex analysis

Polymorphic alleles were identified by heteroduplex analysis. Duplexes were formed by denaturation of 20 µl of PCR product at 95°C for 3 min, followed by cooling to 37°C over 30 min. Samples were separated by shape and size on × 1 MDE polyacrylamide gels (Pharmacia-Hoeffer, UK), in × 0.6 Tris borate buffer (NBL Gene Sciences, UK) containing 18% (w/v) urea, at 600 V for 16 h. DNA present in gels was silver stained using the following protocol: 10% ethanol for 10 min, 1% nitric acid for 10 min, distilled water for 5 min, 0.24% (w/v) silver nitrate for 20 min, distilled water rinse for 10 s. Silver staining was developed in two changes of 230 mM sodium carbonate and 0.05% formaldehyde (37%) and fixed in 0.1 M citric acid.

### DNA sequencing

Samples with variant and invariant banding patterns were DNA sequenced using the Sequenase protocol (Amersham Life Sciences, UK). NAT1-specific sequences were PCR amplified as above, except for substitution of the antisense primer for an identical biotinylated primer. Amplified DNA was purified using the Wizard DNA clean-up system (Promega, UK) and denatured using streptavidin-coated superparamagnetic beads (Dynabeads, Dynal, Norway), both according to manufacturers' instructions. The sense strand was sequenced according to the Sequenase protocol using 0.5 pmol of primer P2 and 5 µCi of [α-$^{35}$S]dATP. Samples were denatured at 95°C for 3 min, then electrophoresed on 6% denaturing polyacrylamide gels at 65 W for 2 h. Gels were fixed in 10% methanol, 10% acetic acid and then dried under vacuum for 2 h. Dried gels were exposed to X-Omat radiographic film overnight and developed automatically in a X2 Hyperprocessor (Amersham, UK). The DNA sequence was determined manually.

### Data analysis

Allele frequencies for each group were calculated and genotype distribution for cancers and controls were tested for goodness of fit with the Hardy–Weinberg equilibrium. Any deviations from this equilibrium were calculated using a chi-squared test. Genotype distributions of the colon cancer and control groups were compared using the chi-squared test incorporating Yates continuity correction, and *P*-values less than 0.05 were considered to be significant.

### RESULTS

### Detection and identification of NAT1 coding sequence polymorphisms

Using NAT1 specific primers, a 225-bp DNA fragment was amplified from 260 colon cancer patient DNAs, and heteroduplex analysis produced a common single band pattern (allele NAT1*4) and a variant pattern of two or three bands on silver stained polyacrylamide gels (Figure 1). DNA sequencing of the variant samples identified two separate base changes, G to A at position 1000 bp (allele NAT1*15) or C to T at position 999 bp (allele NAT1*14) of the NAT1 gene (Figure 2). These base changes result in an amino acid substitution Arg$^{187}$-Gln ( NAT1*15) and a stop

© Cancer Research Campaign 1998

**Table 1** Distribution of NAT1 allele frequency in 260 colorectal cancer cases and 323 control samples

| | NAT1 allele frequency | | |
|---|---|---|---|
| | NAT1*4 | NAT1*15 | NAT1*14 |
| Cancer group | 0.975 | 0.019 | 0.006 |
| Control group | 0.98 | 0.02 | 0 |

**Table 2** Distribution of NAT1 genotype with clinical features of colorectal cancers in 260 colorectal cancer cases and 323 control samples

| | NAT1 genotype | | | |
|---|---|---|---|---|
| | NAT1*4/*4 | NAT1*4/*15 | NAT1*4/*14 | $\chi^2$ analysis |
| Cancer group | 247 | 10 | 3 | |
| Control group | 311 | 12 | 0 | $\chi^2 = 1.84$, $P > 0.3$ |
| *Cancer group only* | | | | |
| Sex | | | | |
| Male | 133 | 3 | 1 | |
| Female | 114 | 7 | 2 | $\chi^2 = 1.14$, $P \geq 0.3$ |
| Side | | | | |
| Right | 86 | 5 | 2 | |
| Left | 161 | 5 | 1 | $\chi^2 = 0.679$, $P \geq 0.5$ |
| Age at presentation (years) | | | | |
| < 70 | 122 | 5 | 1 | |
| ≥ 70 | 125 | 5 | 2 | $\chi^2 = 0.072$, $P \geq 0.5$ |
| Dukes' stage | | | | |
| A | 28 | 1 | 0 | |
| B | 114 | 6 | 3 | |
| C | 105 | 3 | 0 | $\chi^2 = 1.8$, $P \geq 0.5$ |

$\chi^2$ analyses of 2 × 3 and 3 × 3 contingency tables were calculated using Yates correction.

codon Arg[187]-stop (NAT1*14) and were named in accordance with the nomenclature of Vatsis et al (1995). DNA sequence analysis of 22 samples with a single heteroduplex band showed the predicted wild-type sequence.

### NAT1 polymorphism and colorectal cancer

NAT1 allele frequencies and the distribution of NAT1 genotype in 260 cancers and 323 controls are shown in Tables 1 and 2. NAT1 alleles NAT1*4 and NAT1*15 occurred with similar frequencies in the cancer and the control groups, but allele NAT1*14 was present at low frequency (0.006) and found only in the cancer group. For each group, the distribution of genotype was consistent with the Hardy–Weinberg equilibrium (colon cancers $\chi^2 = 0.17$, d.f. = 2, $P \geq 0.5$; control group $\chi^2 = 0.12$, d.f. = 1, $P \geq 0.5$) implying that genotype frequencies are constant and not affected by mutation and selection within each group. Despite the presence of allele NAT1*14 in the cancer group, the distribution of genotype between control and cancer groups was not significantly different (chi-squared using Yates correction = 1.84, d.f. = 2, $P \geq 0.3$) and indicates no selection bias for genotype with colorectal cancer. We found no difference in NAT1 genotype distribution with sex, age of onset, site and Dukes' stage in the cancer group (Table 2).

**Table 3** Distribution of functional polymorphisms in NAT1 and NAT2 genes in colorectal cancers and the control group

| | NAT1 genotype | | |
|---|---|---|---|
| | NAT1*4/*4 | NAT1*4/*15 | NAT1*4/*14 |
| *Colorectal cancer group* | | | |
| NAT2 genotype | | | |
| Predicted fast | 81 (91) | 8 (9) | 0 |
| Predicted slow | 151 (96.8) | 2 (1.3) | 3 (1.9) |
| *Control group* | | | |
| NAT2 genotype | | | |
| Predicted fast | 82 (88.2) | 11 (11.8) | 0 |
| Predicted slow | 123 (99.2) | 1 (0.8) | 0 |

Numbers in parentheses are percentages.

The NAT2 genotype of 217 control and 245 colorectal cancer samples was determined previously (Hubbard et al, 1997). The NAT1 genotype NAT1*4/NAT1*15 was significantly less frequent in individuals who had a slow NAT2 genotype (Table 3). This was observed in both cancer ($\chi^2 = 7.02$, 2 d.f., $P < 0.05$) and control ($\chi^2 = 9.8$, 1 d.f., $P < 0.01$) groups and was unrelated to cancer risk.

### DISCUSSION

We have identified two polymorphisms within the protein coding region of the NAT1 gene. One polymorphism results in an amino acid substitution at codon 187 and is present at a similar frequency in colorectal cancer patients and in control samples. The second polymorphism is present at low frequency and was detected only in the colorectal cancer group. This introduces a stop codon at position 187. Although neither polymorphism is associated with any clinically defined subgroup of colorectal cancer (sex, site of cancer, age of onset and Dukes' stage), the relationship between NAT1 and NAT2 polymorphisms was non-random in both control and cancer groups. This suggests that combined genotypes of NAT1 and NAT2 do not cooperate in cancer risk and indicates either a genetic linkage or a relationship between these enzymes.

Other polymorphisms have been found in the NAT1 gene that cluster around the 3′ untranslated region of the gene (Vatsis and Weber, 1993), one of which is thought to affect enzyme activity (Bell et al, 1995*b*). It is not known whether various polymorphisms in NAT1 are linked or what effect multiple polymorphisms may have on enzyme activity. Biochemical variation in NAT1 enzyme activity has been detected when individual patients were tested using the substrate P-aminobenzoic acid (Vatsis and Weber, 1993; Vatsis et al, 1995), but polymorphisms identified in the polyadenylation region of the NAT1 gene (Vatsis and Weber, 1993; Bell et al, 1995*a*), have failed to account for some slow acetylators, thereby indicating the presence of additional factors influencing NAT1 enzyme activity. The two polymorphisms described in this report occur within the coding sequence and may reduce enzyme activity or alter enzyme specificity. High NAT1 activity has been previously associated with an increased risk of bladder and colorectal cancer (Badawi et al, 1995; Bell et al, 1995*a*), suggesting that NAT1 may play an activation role in carcinogenesis. The precise mechanisms that may be involved remain unclear. Larger study groups may help to clarify the precise nature of cancer risk associated with these polymorphisms, particularly when mutant alleles

© Cancer Research Campaign 1998

are infrequent, and allow analysis of multiple xenobiotic enzyme polymorphisms. However, infrequent alleles are likely to have only a small influence on overall risk.

The substitution of the hydrophilic amino acid glycine for arginine (allele NAT1*15) at codon 187 may affect enzyme activity and specificity, as this domain is thought to be critical for determining substrate specificity (Dupret et al, 1994). The introduction of a stop codon (allele NAT1*14) at codon[187], results in the loss of 104 amino acids from the C-terminus of NAT1. Although the putative active site remains with the truncated protein (residues 47–111), the C-terminus amino acids (residues 211–250) play an important role in enzyme stability, and residues 112–210 determine specificity (Dupret et al, 1994). Therefore, this polymorphism is likely to severely impair or abrogate enzyme function. However, there may only be small differences in enzyme activity between genotypes NAT1*4/*4, NAT1*4/*14 and NAT1*4/*15. This has yet to be demonstrated biochemically. It is interesting to note that allele NAT1*14 was found only in colon cancer patients. The NAT1 genotype NAT1*4/NAT1*15 was less frequent than expected, if random distribution is assumed, in individuals with a slow NAT2 genotype in both the control and the cancer groups. This may represent genetic linkage between NAT1 and NAT2, which are closely related and have overlapping substrate specificity (Turesky et al, 1991). This linkage may confound studies of NAT1 and NAT2 polymorphism with disease susceptibility.

Polymorphisms within the coding region of the NAT1 gene are infrequent and do not appear to be independently associated with colorectal cancer risk. However, these alleles are likely to affect enzyme activity and may be in linkage with particular alleles of the NAT2 gene.

### ACKNOWLEDGEMENT

This work was supported by Cancer Research Campaign grant number SP2288.

### REFERENCES

Agundez JAG, Ladero JM, Olivera M, Abildua R, Roman JM and Benitez J (1995) Genetic analysis of arylamine N-acetyltransferase polymorphism in breast cancer patients. *Oncology* **52**: 7–11

Badawi AF, Hirvonen A, Bell DA, Lang NP and Kadlubar FF (1995) Role of aromatic amine acetyltransferases, NAT1 and NAT2, in carcinogen-DNA adduct formation in the human urinary bladder. *Cancer Res* **55**: 5230–5237

Bell DA, Stephens EA, Castriano T, Umbach DM, Watson M, Deakin M, Elder J, Hendrickse C, Duncan H and Strange RC (1995*a*) Polyadenylation polymorphism in the acetyltransferase 1 gene (NAT1) increases risk of colorectal cancer. *Cancer Res* **55**: 3537–3542

Bell DA, Badawi AF, Lang NP, Ilett KF, Kadlubar FF and Hirvonen A (1995*b*) Polymorphism in the N-acetyltransferase 1 (NAT1) polyadenylation signal: association of NAT1*10 allele with higher N-acetylation activity in bladder and colon tissue. *Cancer Res* **55**: 5226–5229

Blum M, Grant DM, McBride W, Heim M and Meyer UA (1990) Human arylamine N-acetyltransferase genes: isolation, chromosomal localization, and functional expression. *DNA Cell Biol* **9**: 193–203

Cantlay AM, Smith CAD, Wallace WA, Yap PL, Lamb D and Harrison DJ (1995) Heterogeneous expression and polymorphic genotype of glutathione S-transferases in human lung. *Thorax* **49**: 1010–1014

Dupret J, Goodfellow GH, Janezic SA and Grant DM (1994) Structure–function studies of human arylamine N-acetyltransferases NAT1 and NAT2. *J Biol Chem* **269**: 26830–26835

Franke S, Klawitz I, Schnakenberg E, Rommel B, Van de Ven W, Bullerdiek J and Schloot W (1994) Isolation and mapping of a cosmid clone containing the human NAT2 gene. *Biochem Biophys Res Commun* **199**: 52–55

Grant DM, Hughes NC, Janezic SA, Goodfellow GH, Chen HJ, Gaedigk A, Yu VL and Grewal R (1997) Human acetyltransferase polymorphisms. *Mutat Res* **376**: 61–71

Hein DW, Rustan TD, Doll MA, Bucher KD, Ferguson RJ, Feng Y, Furman EJ and Gray K (1992) Acetyltransferases and susceptibility to chemicals. *Toxicol Lett* **64–65**: spec. no. 123–30

Hubbard AL, Harrison DJ, Moyes C, Wyllie AH, Cunningham C, Mannion E and Smith CAD (1997) N-acetyltransferase 2 genotype in colorectal cancer and selective gene retention in cancers with chromosome 8p deletions. *Gut* **41**: 229–234

Lang NP, Chu DZJ, Hunter CF, Kendall DC, Flammang TJ and Kadlubar FF (1986) Role of aromatic amine N-acetyltransferase in human colorectal carcinoma. *Arch Surg* **121**: 1259–1261

Risch A, Wallace DMA, Bathers S and Sim E (1995) Slow N-acetylation genotype is a susceptibility factor in occupational and smoking related bladder cancer. *Hum Mol Gene* **4**: 231–236

Sugimura T, Nagao M and Wakabayashi K (1994) Heterocyclic amines in cooked foods: candidates for causation of common cancers. *J Natl Cancer Inst* **86**: 2–4

Turesky RJ, Lang NP, Butler MA, Teitel CH and Kadlubar FF (1991) Metabolic activation of carcinogenic heterocyclic amines by human liver and colon. *Carcinogenesis* **12**: 1839–1845

Vatsis KP and Weber WW (1993) Structural heterogeneity of Caucasian N-acetyltransferase at the NAT1 gene locus. *Arch Biochem Biophys* **301**: 71–76

Vatsis KP, Weber WW, Bell DA, Dupret J-M, Evans DAP, Grant DM, Hein DW, Lin HJ, Meyer UA, Relling MV, Sim E, Suzuki T and Yamazoe Y (1995) Nomenclature for N-acetyltransferases. *Pharmacogenetics* **5**: 1–7

Vineis P (1994) Epidemiology of cancer from exposure to arylamines. *Environ Health Perspect* **106** (suppl. 6): 7–10

Weber WW and Vatsis KP (1993) Interindividual variability in p-aminobenzoic acid N-acetylation by human N-acetyltransferase (NAT1) of peripheral blood. *Pharmacogenetics* **3**: 209–212

© Cancer Research Campaign 1998